# Recent advances and controversies in diagnosing and treating male infertility

**DOI:** 10.12703/r/22

**Published:** 2020-12-10

**Authors:** Rafael F Ambar, Neel Parekh, Ashok Agarwal

**Affiliations:** 1 American Center for Reproductive Medicine, Cleveland Clinic, Cleveland, OH, USA; 2 Urology Department of Faculdade de Medicina do ABC - FMABC/Andrology Group at Ideia Fertil Institute of Human Reproduction, Santo André, SP, Brazil; 3 Hope Clinic – Fertility Center, Sao Paulo, SP, Brazil

**Keywords:** Home-based sperm testing, Male Infertility, Sperm DNA fragmentation, Proteomics, Varicocele

## Abstract

Interest in male infertility has increased, as it plays an important role in up to 50% of couples struggling with infertility, which is an estimated 48.5 million couples globally. Despite recent advances, diagnosing and treating male infertility remain a significant clinical challenge owing to complex multifactorial pathways and the diversity of treatment options. This review will assess current controversial topics on male infertility such as the use of home-based semen testing, management of subclinical varicocele, and recent advances in the field of sperm proteomics.

## Introduction

Infertility affects roughly 48.5 million couples around the globe^1^, and male factor infertility may play a role in up to 50% of cases, being solely responsible in 20% of cases^2^. The continuous search for methods to improve fertility outcomes in infertile couples, combined with evidence of decline in semen quality among young healthy men worldwide, has increased the focus on male infertility^1,3^. With this expanded interest, many new studies have been directed towards male infertility, with an exponential growth in the number of publications^4^.

In this review article, the most current and significant advances in this field will be addressed, focusing on home semen testing, a better understanding of oxidative stress (OS) and DNA damage testing, new directions in varicocele repair, and a review of the growing impact of proteomics.

## Recent advances in home-based male infertility tests

The study of human reproduction has identified male factor infertility as an important disease that should not be neglected. However, approximately 30% of male partners do not undergo a complete evaluation prior to the utilization of assisted reproductive technology (ART)^5^. Men are more reluctant to seek medical help and frequently struggle with providing a semen sample in a laboratory setting^6^. Furthermore, clinical conditions such as post-vasectomy reversal or patients undergoing chemo/radiotherapy, to name but a few, may require serial semen analyses, making patient compliance even more difficult. Therefore, home-based semen testing has been developed as a convenient solution to these limitations.

**Figure 1.**
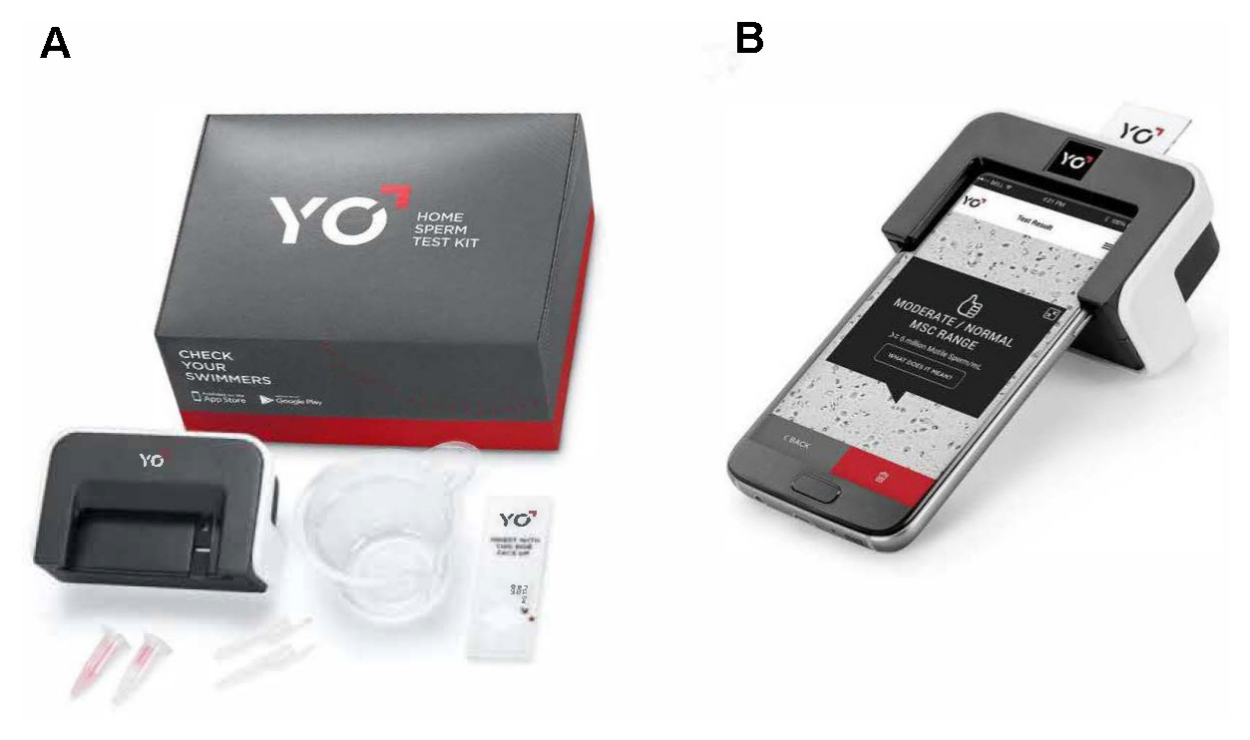
The Yo® Home Sperm Test Kit. **A**: Kit box and contents (clip, cups, slide, liquefaction powder, and transfer pipettes). **B**: assembled YO® clip with inserted testing slide. Reprinted with permission, Cleveland Clinic Center for Medical Art & Photography ©2020. All Rights Reserved.

Currently, there are several home-based semen analyzers approved by the U.S. Food and Drug Administration (FDA). These devices utilize microfluidics in association with smartphone technology, centrifugation technique, or an immunodiagnostic assay^7^. Nonetheless, many of these products only assess fertility potential by measuring sperm concentration, which is only one aspect of the semen analysis. A recent study tested a new smartphone-based sperm test on its ability to provide motile sperm concentration. The authors report that the YO Home Sperm Test (Figure 1) can add motility information to the analysis with a high level of accuracy and precision, allowing it to be a reasonable option for screening purposes^8^. The lack of evaluation of other parameters such as morphology, volume, and pH are clear limitations of these methods, but the user-friendly platforms and ease of access that these smartphone-based devices provide are useful tools in appropriately selected cases^8^. In 2019, a small and easily portable device integrating autofocus optical technology, artificial intelligence (AI) algorithms, electronic engineering, and a mechanical system was studied. The authors have shown that this mini CASA system is reliable and easy to use, providing data on sperm concentration, sperm motility, sperm progressive motility, and seminal pH^9^. Another recent article described the use of a smartphone-based device to assess advanced sperm tests such as the hyaluronic binding assay and sperm DNA fragmentation by the Halosperm test^10,11^. The authors reported high correlation on the parameters analyzed when compared to conventional analysis, although further tests are required for its validation.

Despite the attractive advances in home-based devices, caution should be taken in regard to patient selection, mainly in cases of self-testing, since it may lead to a false sense of security, potentially delaying medical evaluation.

## Advances and controversies on oxidative stress, DNA damage, and antioxidants

Sperm DNA fragmentation (SDF) as well as its causes and impact on successful pregnancies are a frequently discussed topic in the field of male infertility. The increased interest in the use of sperm DNA fragmentation and OS testing for diagnostic or treatment purposes as well as recent evidence discussed below led international societies (American Urological Association and European Association of Urology) to acknowledge the importance of SDF levels in their guidelines^12,13^. In a novel approach, using scientometric analysis, Baskaran *et al*.^14^ showed a linear increasing trend in the number of publications on SDF and ART in the last few decades as well as a larger number of publications concerning clinical scenarios related to SDF. In this section, the role of SDF and its association with OS will be discussed regarding the diagnosis and treatment of male infertility.

### Diagnosis

Recent literature suggests that 30 to 80% of infertile men have elevated seminal reactive oxygen species (ROS) levels. Furthermore, the negative effect of OS and sperm DNA damage on male fertility has also been demonstrated^15,16^. Therefore, in 2019, Agarwal *et al*. proposed the terminology “Male Oxidative Stress Infertility” (MOSI) for patients with abnormal semen characteristics and OS, which includes many patients previously classified as having idiopathic male infertility^17^.

**Figure 2.**
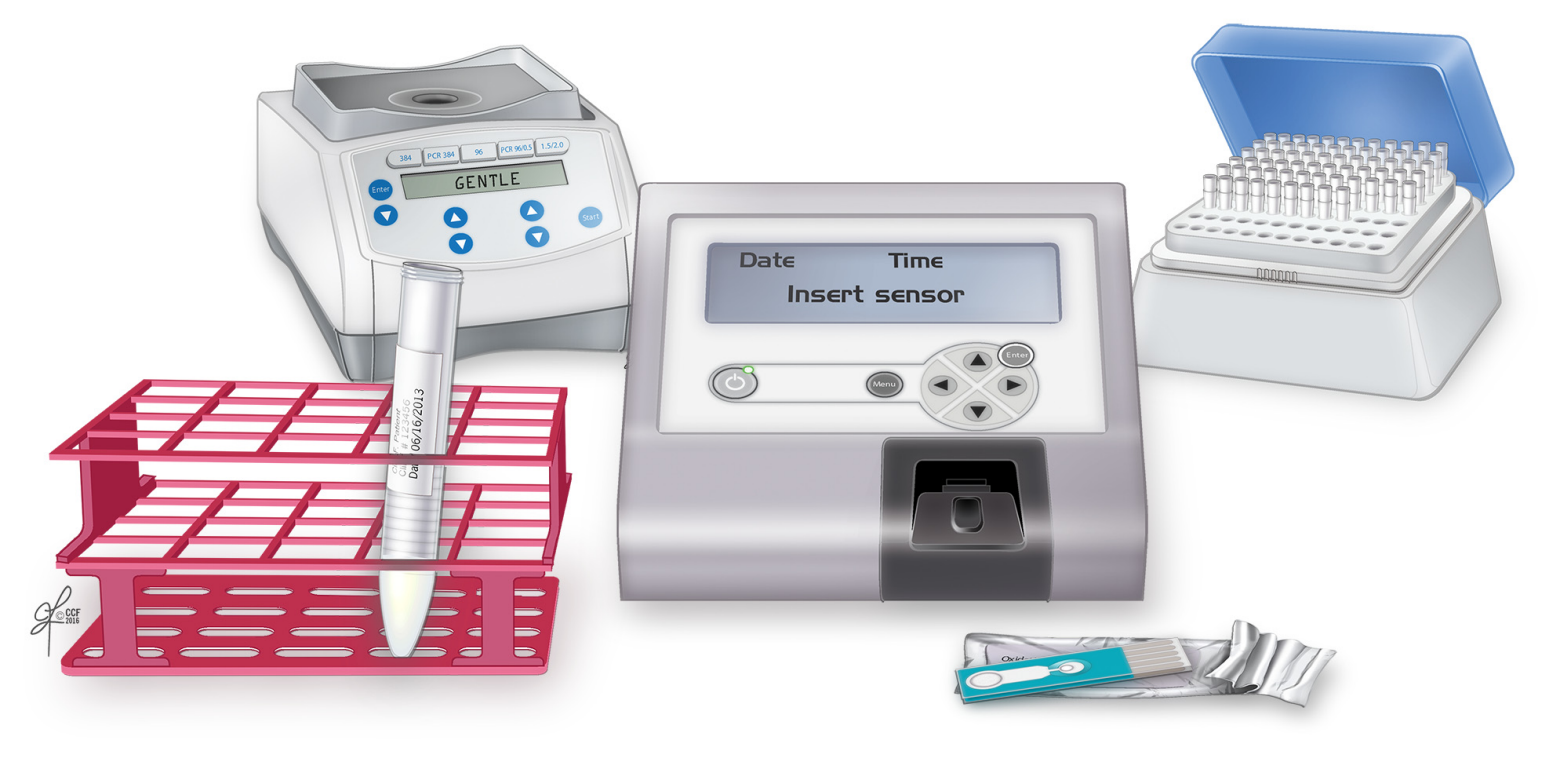
MiOXSYS® analyzer clinical set-up. Reprinted with permission, Cleveland Clinic Center for Medical Art & Photography ©2020. All Rights Reserved.

The measurement of seminal OS can be performed in a variety of ways. Unfortunately, the majority of these tests are expensive and complex and have an extensive learning curve^18^. An easily performed and reproducible alternative is the use of the Male Infertility Oxidative System (MiOXSYS) (Figure 2). This recently developed assay assesses the oxidation-reduction potential (ORP), a useful biomarker of ROS–antioxidant imbalance and, therefore, OS^19^. The validation study for ORP testing using MiOXSYS, published in 2016, demonstrated that ORP provides information on the existing balance between total oxidants and reductants in a biological system (static ORP) and on the measure of antioxidant capacity reserve (cORP). These measures are effective predictors of normal sperm concentration, total count, and motility in infertile men^18^. The results described above have also been validated in a larger, multicenter study, which included 2,092 infertile men, where an ORP of greater than 1.34 mV/10^6^ was found to distinguish patients with normal or abnormal semen quality^19^. The same group conducted a receiver operating characteristic (ROC) analysis and established a cutoff value of 1.42 mV/10^6^ to differentiate fertile from infertile semen groups^20^.

Assays of sperm DNA integrity are examples of advanced sperm function tests that have been extensively investigated over the past decade^21^. Nevertheless, the variability among the different testing methods, such as terminal deoxynucleotidyl transferase dUTP nick end labeling (TUNEL), Comet, sperm chromatin structure assay (SCSA), and sperm chromatin dispersion (SCD), together with the uncertainty of the nature and type of DNA damage delay the use of these assays on a routine basis. A recent review article described the advantages and disadvantages of the most common tests for DNA damage with the cutoff values that have been described for each. Despite the heterogeneity of cutoff values, the authors describe the role of sperm DNA damage in different clinical scenarios, such as varicocele, recurrent pregnancy loss, and unexplained infertility^21^. Furthermore, a recent retrospective study^22^, including more than 600 subjects undergoing intracytoplasmic sperm injection (ICSI), concluded that high DNA stainability (HDS), assessed by SCSA, correlated with early miscarriage (less than 12 weeks) after ICSI. The authors failed to show the same results on *in vitro* fertilization (IVF) cycles, which differs from previous studies^23,24^; however, the difference in SDF testing methods in these studies has to be considered as a cause for contradictory findings.

Finally, a comprehensive meta-analysis published in 2017 demonstrated a significant negative effect of sperm DNA damage on clinical pregnancy following IVF and/or ICSI treatment^25^.

### Treatment

Despite the lack of standard recommendations on the use of DNA fragmentation testing in clinical practice, there is increasing evidence that highlights the importance of this test in male infertility treatment and it will be discussed in this section.

The first step to treat high SDF consists of clinical management, which involves both the treatment of underlying conditions related to sperm DNA damage as well as antioxidant therapy. The most commonly used antioxidants are vitamin C (500–1,000 mg), vitamin E (400 mg), carnitines (500–1,000 mg), coenzyme Q10 (Co-Q10) (100–300 mg), N-acetyl cysteine (600 mg), zinc (25–400 mg), folic acid (0.5 mg), lycopene (6–8 mg), and selenium (200 µg)^26,27^. A Cochrane systematic review and meta-analysis^28^ was published in 2019. In this study, vitamin E and zinc intake were associated with increased pregnancy and live birth rates via natural conception. In a similar study, intake of an antioxidant combination (lycopene 6 mg, vitamin E 400 IU, vitamin C 100 mg, zinc 25 mg, selenium 26 μg, folate 0.5 mg, and garlic 1,000 mg) was demonstrated to increase pregnancy and live birth rate in IVF couples. However, the quality of these results was considered to be low and could not support a recommendation for its use. The multiple combinations among different antioxidants, wide dosage ranges, and large heterogeneity of subfertile men are obstacles that need to be overcome to obtain a definitive conclusion about the optimal preparation to be selected. However, these recent results seem to be a promising way to manage seminal OS and, therefore, decrease SDF^26,27^.

The use of SDF testing was proposed in a clinical practice guideline published in 2017^29^. The authors recommended SDF testing in selected cases of varicocele, unexplained male infertility (UMI), intrauterine insemination (IUI) failure, recurrent pregnancy loss, and IVF or ICSI failure, as seen in Table 1. The goal of these guidelines is to include SDF testing in clinical situations where the management is not uniform in practice but it may help to make a more informed decision.

**Table 1.** Indications of sperm DNA fragmentation (SDF) testing in clinical practice (as seen in Agarwal *et al*.^29^).

Indication	Evidence
**Varicocele** 1. **Patients with grade 2 or 3 varicocele with normal semen parameters** 2. **Patients with grade 1 varicocele with borderline/abnormal semen** **parameters**	- Significant association between SDF and varicocele has been detected - Varicocelectomy reduces percentage of SDF resulting in improved pregnancy rates
**Unexplained infertility/intrauterine insemination (IUI) failure/** **recurrent pregnancy loss (RPL)** 1. **Infertile couples with RPL or prior to initiating IUI** 2. **Early *in vitro* fertilization (IVF) or intracytoplasmic sperm** **injection (ICSI) may be an alternative for infertile couples with** **RPL or failed IUI**	- Infertile men with normal semen parameters can show high SDF - SDF is an independent predictor of male fertility status - SDF levels can predict the likelihood of natural pregnancy - High SDF is associated with lower IUI pregnancy rates - High SDF is associated with higher incidence of abortion
**IVF and ICSI failure** 1. **Patients with recurrent assisted reproductive technology (ART)** **failure** 2. **Use of testicular sperm in non-azoospermic men showing high SDF** **and recurrent IVF failure**	- SDF modestly affects IVF pregnancy rates - SDF does not affect ICSI pregnancy rates - Higher live birth rate is observed in men with low SDF - High SDF is associated with greater incidence of abortion in both IVF and ICSI - Testicular sperm have lower SDF than ejaculated sperm - Increased IVF/ICSI success rate with testicular sperm
**Borderline abnormal (or normal) semen parameters with risk factors** 1. **Patients who have a modifiable lifestyle risk factor of male infertility** **(obesity, smoking, radiation)**	- Modifiable lifestyle risk factors, including smoking and obesity, have a detrimental effect on SDF

One of the most controversial issues concerning SDF is the use of testicular sperm for ICSI in non-azoospermic men with high SDF in the ejaculate and a previously failed ICSI cycle. The use of ejaculated sperm with an elevated level of DNA fragmentation is associated with miscarriage after ICSI^24^. Recent studies have reported that testicular sperm has lower levels of SDF when compared to ejaculate samples^30,31^. These concepts have led to the use of testicular sperm in non-azoospermic patients in an attempt to improve ART results. Furthermore, several series comparing ICSI outcomes using either testicular (T-ICSI) or ejaculated (Ej-ICSI) sperm have reported a significant decrease in miscarriage rates and increase in live birth rates with T-ICSI compared to Ej-ICSI^30,32,33^. Conversely, a meta-analysis conducted by Abhyankar *et al*.^34^ failed to show benefit in the use of testicular sperm, as also found by Awaga *et al*.^35^, who concluded that there is a lack of strong evidence to support this strategy. One of the most common concerns about the use of testicular sperm was the purported higher incidence of aneuploidy compared to ejaculated sperm. However, Cheung *et al*. contested this concept, showing a lower aneuploidy rate in testicular sperm^36^. Despite this, overall evidence seems to indicate a benefit with the use of testicular sperm in this scenario, but additional research is required in order to change practice patterns^37^.

## Advances and controversies in varicocele and male infertility

### Varicocele and assisted reproductive technology outcomes

The pathophysiology of varicocele is not completely understood, but emerging evidence on the impact of varicocele treatment shows the importance of this condition in patients with male infertility. A meta-analysis conducted by Esteves *et al.* showed that varicocelectomy performed before ICSI may improve clinical pregnancy and live birth rates in men with clinical varicocele^38^. A review conducted by Kohn *et al*. has also summarized the benefit of varicocele treatment on IVF and ICSI outcomes. However, the cost-effectiveness of this approach should be better evaluated^39,40^.

### Varicocele and non-obstructive azoospermia

A meta-analysis^41^, published in 2016, addressed the benefit of varicocele treatment in patients with non-obstructive azoospermia (NOA). This study showed a higher sperm retrieval rate (OR: 2.65; 95% CI: 1.69–4.14; *P* <0.0001) in patients who underwent varicocelectomy prior to ICSI. The authors also found that 43.9% of patients who underwent varicocelectomy had sperm in their ejaculate after a period that varied from 4.5 to 11 months. These findings are in agreement with previous reports^42,43^, but sperm retrieval is still needed in many cases owing to a low concentration of usable sperm for ICSI^44^. Furthermore, other factors such as female age may also affect the final outcome^42^. Given the possible benefit of this strategy, the European Association of Urology included in their guidelines, since 2017, a statement that varicocelectomy has a role in the improvement of semen parameters, even in patients with NOA^13^.

### Impact of the treatment of subclinical varicocele

In the last few years, the treatment of subclinical varicocele has garnered increased discussion. While the current recommendation is that subclinical varicocele should not be treated, recent studies have advocated the contrary. A retrospective study was performed on 190 infertile men who underwent microsurgical varicocelectomy. The authors demonstrated that patients with clinical or subclinical varicocele had an improvement in total motile sperm count, which could potentially reduce the need for ART or have success with less-aggressive forms of ART^45^. Another group studied 358 infertile men with left clinical and right subclinical varicocele. Patients randomly underwent bilateral or unilateral surgery. A significant improvement in sperm concentration, morphology, and progressive motility as well as spontaneous pregnancy rate was observed in patients who underwent a bilateral procedure^46^. A recent meta-analysis also described a greater increase in pregnancy rates in men who underwent bilateral varicocele repair than men who had unilateral surgery. This result was applicable for clinical bilateral varicocele as well as left clinical varicocele and right subclinical varicocele^47^. Of note, the diagnosis of clinical and subclinical varicocele may vary because of differences in performing the physical exam, and this variability may explain the findings of the abovementioned studies.

## Recent advances in proteomics

Proteomics has been widely applied to the field of human reproduction and, when combined with bioinformatics analysis, appears to be a promising domain to be explored. Research on sperm and the seminal plasma protein profile has expanded extensively over the last few years. The investigation of the differential expression of sperm proteins may help in understanding the molecular pathways implicated in male infertility^48^.

The sperm proteome consists of a total of 6,198 proteins, while 2,064 proteins have been reported in seminal plasma^49,50^. Dysregulation of protein tyrosine phosphatase, non-receptor type 14 (PTPN14), a tyrosine phosphatase protein involved in the regulation of sperm motility, has been reported in asthenozoospermic patients^51^. Oligoasthenozoospermia is also reported to have specific proteome abnormalities, such as downregulation of cystatin 3 (CST3) and upregulation of KLK3 and SEMG1 sperm proteins^52^. A recent proteomics study reported 162 differentially expressed proteins (DEPs) in semen samples of men diagnosed with UMI compared with fertile control patients. This study also suggested that SPA17, ANXA2, and SERPINA5 may potentially serve as non-invasive protein biomarkers associated with the fertilization process of the spermatozoa in UMI patients^53^. Varicocele, as an important condition related to male infertility, has also been studied. Agarwal *et al*. reported that the sperm proteome profile in varicocele patients was able to decipher the subcellular role of proteins responsible for infertility associated with bilateral varicocele^54^. The same group assessed the sperm proteomic profile of varicocele patients and showed that 87% of DEPs involved in sperm function and energy metabolism were downregulated in both unilateral and bilateral varicocele cases^55^. Proteomics is a large field for study and may provide, in the future, useful biomarkers for diagnosis and therapeutics in male infertility.

## Conclusion

Male infertility is a prevalent clinical condition that affects many couples worldwide. Accordingly, research within the field continues to grow, with this review summarizing recent data regarding home semen testing, oxidative and sperm DNA damage, and the utility of varicocelectomy in various clinical scenarios. Sperm proteomics is a burgeoning area of research which may help elucidate new causes of male infertility in the future.
